# Comparative study of physical-chemical properties of bioactive glass ionomer cement

**DOI:** 10.1590/0103-6440202405728

**Published:** 2024-03-22

**Authors:** Nádia Buzignani Pires Ramos, Klissia Romero Felizardo, Sandrine Bittencourt Berger, Ricardo Danil Guiraldo, Murilo Baena Lopes

**Affiliations:** 1 Department of Restorative Dentistry, School of Dentistry, University of North Parana(UNOPAR). Londrina, Paraná Brazil; 2 Department of Restorative Dentistry, School of Dentistry, University Paranaense(UNIPAR). Umuarama, Paraná Brazil; 3 Department of Restorative Dentistry, School of Dentistry, University Anhanguera-Uniderp(UNIDERP). Campo Grande, Mato Grosso do Sul Brazil

**Keywords:** Glass ionomer cement, Physical properties, Surface properties, Bioactive material

## Abstract

This study analyzed the physical-chemical properties of bioactive ionomer materials. Cention N bioactive materials were evaluated chemically activated (CN) and light-cured (CN-LC), Equia Forte Fill (EQUI); conventional resin composite Filtek Z350 XT (Z350); resin glass ionomer cement Riva light Cure (RIVA) and flowable resin composite Filtek Bulk Fill Flow (BULK-F) were evaluated. Sixty specimens (n=10) were prepared for sorption (SR), solubility (SL), flexural strength (FS), shrinkage stress (SS), conversion degree (CD), microhardness (MI), and surface roughness (SR) tests. Non-cured and light-cured materials were assessed on FTIR. 30 human molar teeth were used in the bond strength test (BS). Data were subjected to ANOVA and post-hoc Tukey’s test (5% of significance). EQUI showed more sorption in SR and no statistical difference from RIVA and CN-LC. CN group showed more solubility and EQUI presented less (p<0.05). BULK-F showed higher FS (MPa), without differences from CN and Z350, whereas EQUI presented the lowest FS not differing from RIVA. BULK-F and CN-LC showed more shrinkage stress differing from EQUI. CN-LC and CN showed higher CD differing from the other which showed no differences (p>0.05) between them. EQUI showed the highest hardness (p<0.05) in MI. There were no differences (p>0.05) in SR (µm). Z350 and BULK-F presented higher BS, whereas CN-LC showed the lowest, although not differing from EQUI and RIVA. Equia Forte's solubility and microhardness make it a good alternative as a restorative material. Cention N degree of conversion and flexural strength making it an esthetic option to amalgam.

## Resumo

Este estudo analisou as propriedades físico-químicas dos materiais de ionômero bioativo. Os materiais bioativos Cention N quimicamente ativados (CN) e fotopolimerizados (CN-LC), Equia Forte Fill (EQUI); resina composta convencional Filtek Z350 XT (Z350); resina de ionômero de vidro Riva light Cure (RIVA) e resina composta fluida Filtek Bulk Fill Flow (BULK-F) foram avaliados. Sessenta espécimes (n=10) foram preparados para testes de sorção (SR), solubilidade (SL), resistência à flexão (FS), tensão de contração (SS), grau de conversão (CD), microdureza (MI) e rugosidade da superfície (SR). Os materiais não curados e curados com luz foram avaliados por FTIR. 30 dentes molares humanos foram usados no teste de resistência de união (BS). Os dados foram submetidos a ANOVA e teste de post-hoc Tukey (5% de significância). O EQUI apresentou maior sorção no SR e não mostrou diferença estatística em relação aos grupos RIVA e CN-LC. O grupo CN apresentou maior solubilidade e o EQUI apresentou menor (p<0,05). O BULK-F apresentou maior FS (MPa), sem diferir do CN e do Z350, enquanto o EQUI apresentou o menor FS, não diferindo do RIVA. O BULK-F e o CN-LC apresentaram maior estresse de contração, diferindo do EQUI. O CN-LC e o CN apresentaram maior CD (%), diferindo dos demais, que não apresentaram diferenças (p>0,05) entre si. A EQUI apresentou a maior dureza (p<0,05) no MI. Não houve diferenças (p>0,05) na SR (µm). Z350 and BULK-F apresentaram BS, enquanto a CN-LC apresentou a menor, embora não diferindo da EQUI e da RIVA. A solubilidade e a microdureza do Equia Forte o tornam uma boa alternativa como material restaurador. O grau de conversão e a resistência à flexão do Cention N o tornam uma opção estética ao amálgama.

## Introduction

Glass ionomer cement are versatile material used in dentistry as restorative, base, or liner materials due to its features such as biocompatibility, fluoride release, chemical adhesion to dental tissue, and low toxicity ^1^ ^,^ ^2^ ^,^ ^3^. However, ionomer cement presents some clinical disadvantages, such as fragility and initial sensitivity to humidity, a fact that can reduce restoration life span. Another disadvantage is its sorption and solubility, which can lead to restoration losses due to fracture and marginal infiltration ^4^.

The search for more reliable materials, with more esthetical and durability features, gave birth to bioactive materials. Equia Forte is a restoration system composed of 3 products: glass ionomer cement added with highly reactive glass particles, high molecular weight polyacrylic acid, and resin protector. Based on the manufacturer, the viscosity of the original ionomer, associated with a layer of nanometric charge, based on a more reactive silica, improves its mechanical properties and handling ^5^. Cention N was launched as a radiopaque, self-curing restoration material with a light cure option; it belongs to the group of alkasite-type resin composites ^6^, which are composed of three glass types, besides having ytterbium fluoride. Calcium fluorosilicate is the active glass aimed at increasing the release of H+ Ca2+ and F- ions; it helps re-mineralize tooth enamel by releasing fluoride ions at acidic and neutral pH.

Bioactive materials emerged as alternatives to traditional composites and glass ionomer cement that, although releasing a significant amount of fluoride, present low esthetical resolution and some undesirable physical properties ^7^. These materials closely interact with both biological tissue and the buccal cavity; they actively participate in saliva and the dental structure, within a continuous mineral-exchange cycle. Thus, calcium and phosphorus ions are released from the dental surface; they are available for interaction with the fluoride found in the saliva due to pH increase. These ions can make ionic exchange by carrying and releasing their ionic components, and by returning to their initial state after such stimuli are removed. This process helps achieve a biodynamic balance between teeth and saliva, as well as contributes to maintaining buccal health.

Some materials known as bioactive can improve caries resistance, which may occur due to ionic exchange between the material used and the tooth surface. Besides the action of fluoride found in these materials, it is capable of inducing lower bacterial adhesion and lower incidence of recurrent caries ^8^. Dental restoration materials are subjected to different forces in the buccal cavity, mainly during mastication. Therefore, it is interesting to evaluate the physical and mechanical properties of these materials and compare them to traditional materials, such as resin composites and glass ionomer cement. This study aimed to analyze the physical-mechanical properties of bioactive ionomer materials Cention N and Equia Forte. These hypotheses tested were that each physical-mechanical properties: sorption and solubility, flexural strength, shrinkage stress, conversion degree, micro-hardness, surface roughness, and microshear bond strength were the same among the bioactive glass ionomer cement studied.

## Materials and Methods

The present experimental study was carried out *in vitro* and assessed the physical-mechanical properties of five restorative materials (Box 1). Cention-N was used in its photo-activated (CN-P) and chemically activated (CN) forms. VALO curing light device (Ultradent Products Inc., South Jordan, UT, EUA), at an irradiance of 1200 mW/cm^2^, for 20s, was used for materials requiring photo-activation. All specimens and tests were made by one operator, which was previously calibrated to manipulate the materials. A minimum total specimen size of 54 (ie, n=2) specimens was established with Minitab Software version 17, using an analysis of variance (ANOVA) model, alpha error of 0.05, power of 95%, assumed standard deviation of 15.91, the maximum difference of 142.3. The effect size was hypothesized based on prior related work ^9^. All tests were running with n=10.

**Box 1:**
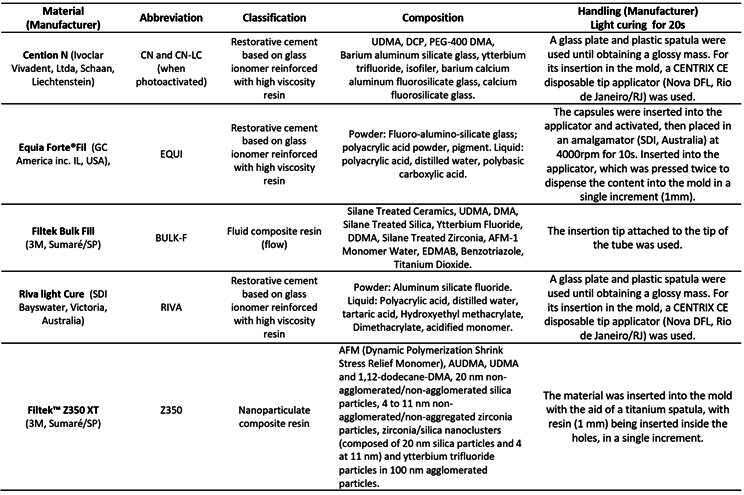
Composition of the materials tested

### Sorption and Solubility

Sixty specimens were prepared with 1mm in thickness and 10mm in diameter (ISO 4049:2019) using a rubber matrix, and divided into 6 groups, based on the used materials (n=10). The specimens were stored for 24h in the dark and after polished with sandpaper #600 for 30s and measured with the aid of a digital caliper (Mitutoyo, Sul Americana, São Paulo, Brazil). The specimens were weighed on a decimal digital scale (Bl3200b, Bioscale, Campinas, SP, Brazil) and stored in individual cap open containers in silica gel desiccator - in the bacteriological stove (ECB2, Biodont, Odontobrás, Ribeirão Preto, SP, Brazil), under controlled temperature (37°C±1°C). The specimens were weighed every 24h until reaching a total mass loss not higher than 0.1mg (m1). Subsequently, the specimens were immersed in 1.5mL of distilled water, sealed, and stored in the stove at 37° C ± 1 ºC, for seven straight days as re-weighted (m2) Next, they were once more stored in their flasks and placed in desiccator, where they remained until the constant mass was found before being measured again (m3). SR and SL values were calculated in milligrams per cubic millimeter (mg/mm^3^). The data was obtained using the equations according to the ISO4049:2019 standard ^10^.

### Flexural strength

Ten specimens (10x2x2 mm) of each material were made using a rubber matrix. The materials were inserted into the matrix and photoactivated using a light cure unit (VALO) according to the manufacturer’s instructions, except by Cention N in chemical mode (CN). The specimens were stored for 24h at 37^o^C in an oven. Then, the flexural strength test was done in a disposite with 8 mm of span size in a universal testing machine (DL2000, Emic, São José dos Pinhais, PR, Brazil) at 0.5 mm/min. Flexural strength (in MPa) was calculated using the equation 3PL/2bh², where P is the maximum fracture load (N), L is the distance between support points (mm), b is the sample width (mm), and h is the sample height (mm).

### Shrinkage stress

Sixty specimens of photoelastic resin (PL3 Vishay, Micro-Measurements Group Inc, Raleigh, NC, USA) with 2mm in height and 20 mm in external diameter with an internal cavity of 5mm in diameter were made using a rubber matrix. The materials to be tested were inserted in the internal cavities of the rings, covered by a Millar strip, and polymerized according to the manufacturer's instructions. Specimens were flattened with aluminum oxide abrasive discs (Norton, Saint-Gobain, Guarulhos, SP, Brazil) and subjected to photoelastic analysis in polariscope (Vishay LF/Z-2, Malvern, USA). Readings were carried out in two opposite points. Pictures of each assay and shrinkage stress measurements (MPa) were recorded by the equipment’s software (PS Calc 3.1). The shrinkage stress was calculated using the equation ε1 - ε2 = Nnλ/2kt, where ε1 - ε2 = principal strains in the coating, Nn = normal-incidence fringe order, λ = wavelength of yellow light (22.7μin, or 575 nm), t =thickness of PL3 resin, k = strain-optic coefficient of coating, f = λ/2kt = fringe value of coating.

### Conversion degree

Conversion Degree (CD) was analyzed in Fourier transform infrared spectroscopy (FTIR) (Frontier, Perkin Elmer, USA). Non-cured materials were placed right on the Attenuated Total Reflectance (ATR) and the data was recorded. Specimens were light-cured based on the manufacturer’s recommendations ground to a grade of agate and placed over ATR. Five spectra were obtained under room temperature conditions (25°C), with 64 screenings with resolutions ranging from 1500-1800 cm^-1^ to 4 cm^-1^. CD was assessed by using aliphatic and aromatic bands, C=O, located in 1716 cm^-1^ and C=C in 1608 cm^-1^, respectively. The aliphatic:aromatic peak ratio was assessed in the non-cured and cured samples. CD was calculated based on the following ratio: CD (%) = {1 - [(Abs(aliphatic) / Abs(aromatic) polymer] / [(Abs(aliphatic) / Abs(aromatic) monomer]} X 100.

### Micro-hardness

The same specimens from the shrinkage stress test were used in this test (5mm in diameter and 2mm in height), totaling 60 specimens. Knoop micro-hardness evaluation (MI) was made on a microhardness testing machine (Shimadzu HMV - G 21S, Barueri, SP, Brazil). Three ^3^ indentations were made on each surface by using a force of 0.25g for 5s. The values were recorded and analyzed by the device’s software. The following formula was used: MH = load/impression area (mm^2^) = P/C_p_/L^2^, where L is the length of indentation along its long axis, Cp is the correction factor related to the shape of the indenter, ideally 0.070279 P is the load.

### Surface roughness

The same specimens from the shrinkage stress test (5mm in diameter and 2mm in height) were used in the surface roughness (SR) test, totaling 60 specimens. The analysis was made on a Surface Roughness Tester (SJ 410, Mitutoyo, Tokyo, Japan) to measure mean roughness (Ra). The analyzed area was at different sites of the micro-hardness test. Three readings were carried out per specimen at a limited displacement from the tip of the roughness meter (0.25 mm), at a speed of 5mm/s, and the data was recorded. The following formula was used:

$$Ra=\int_{O}^{L}/Z(x)/dx$$

Where L = evaluation length and Z(x) = the profile height function.

### Microshear Bond Strength

Thirty human molar teeth were selected and stored for 7 days in 0.5% Chloramine-T solution, cleaned with Mccall periodontal curettes and pumice stone prophylaxis. The teeth used were extracted for orthodontic reasons. The project was approved by the ethics committee under #57359122.5.0000.0108 The presence of hypoplasia, hypocalcification, caries, restorations, bracket resin, teeth with prior bleaching, and enamel fractures was used as an exclusion criterion. The selected teeth had their roots sectioned with a double-sided diamond saw (KG Sorensen, Cotia, SP, Brazil). The crowns were split in half in a mesiodistal direction. Specimens were embedded with acrylic resin (Artigos Odontológicos Clássico Ltda, São Paulo, SP, Brazil) in a PVC ring (Tigre Tubos e conexões, Rio Claro, SP, Brazil) in a way that the buccal, lingual, or palatal aspect of the dental crown be set at 90° to the base. The specimens were polished with 400 and 600-grit aluminum oxide sandpaper (3M, Arotec S/A Ind. e Comércio, Cotia, SP, Brazil). The specimens were subjected to micro-hardness and roughness analysis (baseline) and specimens with values lower and higher than 10% of the mean were excluded from the study. Tygon matrices (Saint-Gobain Performance Plastics, Ohio, EUA) of 0.7mm in diameter and 1.2mm in height were used. The materials were inserted in the tygon tube and placed on the specimen’s dentin surface, which was previously prepared according to the manufacturers’ recommendations. At least two and a maximum of three tigons were applied to each tooth. CN and RIVA have no surface treatment. in the EQUI group, the dentin surface was treated for 10 seconds with conditioner material Cavity, which was provided by the manufacturer. Dentin surface in Z350 and BULK-F groups was treated with 37% phosphoric acid gel (3M Espe, St. Paul, MN, USA), for 15 seconds, and then washed; Adper Single Bond Plus (3M Espe, Sumaré, SP, Brazil) was applied after teeth drying and photo-curing for 10 seconds with VALO (Ultradent Products Inc., South Jordan, UT, USA). The specimens were, then, stored in distilled water, under 100% relative humidity, at 37°C, for 24 hours. The specimens were subjected to a microshear test on the universal testing machine (DL 2000, EMIC, São José dos Pinhais, PR, Brazil) with a 5kg load cell at a speed of 1mm/min, and the values were recorded. The mean of each tooth was made for the statistical analysis. The data was recorded in N, and σt was calculated (in MPa) with the equation: F/A, where F is the maximum fracture resistance, and A is the cross-section area (mm^2^).

The fracture patterns of the specimens were analyzed using a stereomicroscope (STM PRO, Bel Photonics, Italy) with a magnification of up to 6x. The fracture patterns were analyzed by a previously trained professional and classified into Adhesive fracture (A) - when most of the fracture occurs in the adhesive area; Cohesive fracture in the dentine (CS) - when the fracture occurs only in dentin; Cohesive fracture in the composite (CC) - when the fracture occurs in the restorative material, even if incomplete (chip); Mixed fracture (M) - when it occurs in the adhesive area, but still has a large composite remnant.

### Statistical Analysis

The collected data were analyzed for normal distribution by the Kolgomorov-Smirnov test and subjected to one-way analysis of variance (one-way ANOVA) and the differences between groups by Tukey’s test. The significance level was set at 5%.

## Results

All results are presented in Table 1.

**Table 1:** Test results according to the analyzed groups

Groups	Sorption ((g/ml)	Solubility ((g/ml)	Flexural strength (MPa)	Shrinkage stress (MPa)	Conversion degree (%)	Knoop Microhardness (KHN)	SSurface Roughness (µm)	Bonding Strength (MPa)
CN-LC	29,81±8,75 abc	10,70±9,93 bc	57,42±13,35 b	42,30±6,44 a	80,91±1,32 a	58,41±5,77 bc	0,12±0,03 a	2,83±1,58 b
CN	27,13±12,70 bc	25,86±9,40 a	69,97±9,70 ab	39,35±2,64 ab	84,76±3,45 a	67,75±9,84 b	0,12±0,02 a	0,00±0,00 c
EQUIA	42,29±13,57 a	-4,97±7,37 d	14,29±7,58 c	33,20±10,4 b	52,78±1,76 d	95,82±5,89 a	0,14±0,03 a	4,99±1,77 b
BULK-F	16,05±10,25 c	-1,66±11,7 cd	75,67±10,44 a	42,60±5,63 a	33,74±1,18 e	52,64±7,99 c	0,14±0,04 a	17,70±2,68 a
RIVA	33,38±8,66 ab	15,92±10,58 ab	21,45±6,91 c	36,35±2,74 ab	63,55±2,72 c	51,04±12,5 c	0,12±0,02 a	5,82±0,78 b
Z-350	20,64±8,59 bc	-3,95±7,14 d	64,71±18,59 ab	37,85±7,99 ab	75,97±3,28 b	68,16±8,90 b	0,11±0,01 a	15,31±3,36 a

### Sorption and Solubility

EQUI (42.29±13.57 (g/ml) showed more sorption (p<0.05) than other groups, except by CN-LC (29.81±8.75) and RIVA (33,38±8,66); Group BULK-F (16.05±10.25) showed lower sorption, but without differing from groups Z350 (20.64±8.59), CN (27.13±12.70) and CN-LC (29.81±8.75). CN (25.86±9.40) showed higher solubility, but without differing from RIVA (15.92±10.58). EQUI (-4.97±7.37) showed lower solubility, but without differing from Z350 (-3.95±7.14) and BULK-F (-1.66±11.77).

### Flexural strength

Group BULK-F (75.67±10.44 MPa) showed more flexural strength, but without differing from groups CN (69.97±9.70 MPa) and Z350 (64.71±18.59 MPa). Group EQUI (14.29±7.58 MPa) showed lower flexural strength without differing from RIVA (21.45±6.91 MPa).

### Shrinkage stress

Groups BULK-F (42.60±5.63 MPa) and CN-LC (42.30±6.44 MPa) showed more tension (p<0.05) than EQUI (33.20±10.39 MPa), without differing (p>0.05) from the others.

### Conversion Degree

**Figure 1:**
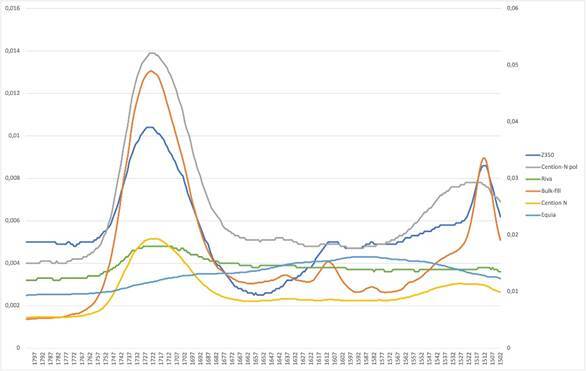
FTIR graph of polymerized materials

Groups CN-LC (80.91±1.32) and CN (84.76±3.45) showed a higher conversion degree (p<0.05) compared to the others. BULK-F (33.74±1.18) showed the lowest conversion. All other materials differed (p<0.05) from each other. The graph for the polymerized materials is shown in Figure 1.

### Micro-hardness

EQUI (95.82±5.89) showed higher hardness compared to the other groups (p<0.05). RIVA (51.04±12.50) and BULK-F (52.64±7.99) showed lower hardness (p<0.05) than the other groups, except for CN-LC (58.4.1±5.77 KHN). CN-LC, CN (67.75±9.84), and Z350 (68.16±8.90 KHN) did not differ from each other (p> 0.05).

### Surface roughness

All materials presented similar roughness (p>0.05).

### Bond strength

Filtek Bulk Fill (17.70±2.68) and Z350 (15.31±3.36 MPa) showed higher Bond Strength (p<0.05) compared to the others. CN (0.00±0.00) was unable to be tested, and all specimens were debonded during 24h storage. The failure analysis is presented in Table 2.

**Table 2:** Failure pattern (%)

	Adhesive (A)	Cohesive in dentin (CS)	Cohesive in composite (CC)	Mixed (M)	Pre-test failure
CN-LC	0 (0%)	0 (0%)	0 (0%)	0 (0%)	10 (100%)
CN	9 (90%)	0 (0%)	0 (0%)	1 (10%)	0 (0%)
EQUIA	8 (80%)	1 (10%)	0 (0%)	1 (10%)	0 (0%)
BULK-F	2 (20%)	2 (20%)	0 (0%)	5 (50%)	0 (0%)
RIVA	6 (60%)	3 (30%)	0 (0%)	1 (10%)	0 (0%)
Z-350	1 (10%)	1 (10%)	0 (0%)	7 (70%)	0 (0%)

## Discussion

The hypothesis that the physical-mechanical properties: sorption and solubility, flexural strength, shrinkage stress, conversion degree, micro-hardness, surface roughness, and microshear bond strength are the same between the bioactive glass ionomer cement studied was all rejected, except by surface roughness.

Water sorption leads to external movement of residual monomers and ions, causing solubility that can trigger failure of the restoration and reduction of mechanical properties, decreasing the durability of resin composites and formation of micro-cracks ^11^. Equia Fort, Riva, and Cention-N when light cured showed more sorption than Filtek Bulk-Fill, which is expected since this material is a polymeric material with high filler content. The manual handling of glass ionomer cement is a factor capable of influencing the sorption of liquids. It can lead to porosity formation and, consequently, increase the liquid sorption in the material ^12^. However, there were no differences in the glass ionomers cement in this study, showing that the calibrated operator could overcome the constraint of manual mixing. After the setting time, desiccation and the consequent loss of water can lead to surface cracking, which can occur because glass ionomer cement absorbs and loses water more easily than resin materials ^13^, as shown by the sorption results for materials Equia Forte, Riva, and Cention N.

Equia Forte and Filtek Z350 presented lower solubility being more favorable in this regard. This finding corroborates to Matick et al ^14^, which showed Equia Forte did not present significant solubility variation over time. Material composition plays an important role in their physical-chemical properties because differences in glass ionomer cement compositions determine the behavior of the materials. According to Farias et al. ^15^, Cention N presented moderate viscosity and strong mechanical properties, hydrophobicity and low water absorption Photo-activated Cention N showed better solubility results than when not activated. This finding assumingly resulted from the higher density of the formed polymeric chain ^16^, as there was no difference in conversion degree or the other evaluated properties.

Equia Forte showed lower flexural strength values but like RIVA. Mishra et al. ^17^. found that Cention N and Equia Forte showed flexural strength reduction over time with lower values than Vitremer. According to Moshaverinia et al. ^18^, Equia Forte showed higher flexural strength than Fuji IX and ChemFil Rock. Cention N showed higher results compared to Fugi IX and Ketac and Riva ^9^; it also presented highly significant values in comparison to the other assessed materials. Chole et al. ^19^ observed higher flexural strength values for Cention N than for resins Ivoclar Tetric N Ceram bulk-fill and nanofill, and glass ionomer cement Fuji IX. Flexural strength tests carried out by Balagopal et al. ^20^ showed that Cention N presented higher flexural strength than Fuji IX due to its highly reticulated polymeric structure accounted for its flexural strength.

This study corroborates to Mishra et al. ^17^ which showed that Cention N flexural strength did not differ from the studied resins. A good explanation for Cention N behavior may lie in the fact that it does not have Bis-GMA (bisphenol A-glycidyl methacrylate), HEMA (2-hydroxyethyl methacrylate), or TEGDMA (triethylene glycol dimethacrylate), being UDMA (urethane-dimethacrylate) its main monomeric matrix component ^15^. Alkasite resins present stable flexural strength, their values are higher than 100MPa, and this is an important factor for restoration longevity since it supports the high stress generated by mastication in the posterior region ^17^ ^,^ ^19^. This could be explained by these resins’ composition: barium aluminum glass, calcium barium aluminum fluoride silicate glass, ytterbium trifluoride, and isophillers. The cured material can release Ca2+, F-, and OH- ions due to environmental acidity, which mineralizes the enamel ^15^. Cention N presented results similar to Z350 and Filtek Bulk Fill. High flexural strength is justified by the polymer’s highly reticulated structure. 

Filtek Bulk Fill and Light Cured Cention N presented higher shrinkage stress than group Equia Forte. Materials with larger inorganic content are expected to present lower volume shrinkage. The higher the molecule weight of a single monomer unit, the lower the volumetric shrinkage. Most conventional materials have dimethacrylate monomers in their composition, however, Z350 has special stress-relieving monomers ^21^, such as high molecular weight aromatic urethane dimethacrylate (AUDMA), which reduces the number of reactive groups in resins, which mitigates volumetric shrinkage and polymeric matrix stiffness.

Shrinkage stress magnitude depends not only on the conformity of surrounding areas but also on the nature of the shrinkage material, mainly on its viscoelastic properties. Stiffer materials are associated with greater tension. This can be applied to Equia Forte, as the lower conversion degree led to a more flexible material, decreasing the shrinkage stress. Material shrinkage stress depends on factors such as charge type and size. Monomer system and light cure also affect shrinkage behavior as determine the material’s polymeric structure ^22^.

The monomeric conversion degree was assessed through Fourier transform infrared spectroscopy (FTIR), based on the attenuated total reflectance (ATR), calculating the conversion degree by comparing the association between aliphatic peak C=O at 1716 cm^-1^ and aromatic C=C peak at 1608 cm^-1^. Cention N was the material presenting more conversion degree and Filtek Bulk Fill was the one presenting less. In methacrylates, polymerization occurs with the opening of a carbon-carbon double bond, leaving a free valence available for reaction with other monomers ^23^. Differences in the ratios of specific monomers, in their re-activity, temperature, mix viscosity, and charge differ from light cure and favor, or limit, the reactions. Since these are materials with different bases, ionomeric or resinous, it was expected that the values would be different and for this very reason, the comparison would serve to understand the results of the other tests performed.

Resins with larger volumes of filler particles present higher micro-hardness values and are influenced by material type ^15^. Alkasite resins showed a filler rate ranging from 12% to 40% of the final material mass. Equia Forte herein presented a higher microhardness value, compared to groups Riva and Filtek Bulk Fill. Chowdhury ^24^ evaluating the hardness found that resin composite, Cention N, silver amalgam, and ionomer can resist the masticatory forces and Cention N showed higher microhardness, becoming an adequate alternative and less invasive treatment.

Surface features are significant factors for the clinical success of any restoration ^25^. Surface roughness and imperfections make restoration more susceptible to dental plaque deposition, gingival irritation, poor esthetics, and prognosis. The material type did not influence roughness values in the present study. All specimens were prepared by using the polyester mylar strip, but they were further polished to eliminate the outcropped matrix.

Significant variations among materials regarding surface wear and roughness were reported to have happened due to the combination of factors such as matrix nature and property, glass ratio, and dimensions in organic particles. Equia Forte is a hybrid glass material without a resin component, which contributes to improved surface roughness in comparison to modified glass ionomer cement ^25^. The fatigue test was herein tested, and it may have contributed to finding no roughness differences among materials.

Light Cure Cention N showed lower bond strength compared to the other groups, however without statistical difference from Equia Fort and Riva. The lower values recorded for ionomers corroborate with Calvo et al. ^26^, which showed that adhesion to dentin occurs through hydrogen bonding to collagen; combined with ionic bond to apatite inside the dentin structure, causing low tensile strength. Feiz et al. ^27^ observed that the bioactive material GIOMER accounted for better bond strength results in deciduous teeth than Cention N. Manuja et al. ^28^ concluded that Fuji IX GIC (glass ionomer cement) presented a lower mean value for shear strength among all tested groups. Studies showed lower GIC adhesion performance in comparison to resin composite because its self-adhesion to dental tissue derives from an ionic exchange mechanism, according to which, polyacrylate ions replace phosphate ions on the surface of hydroxyapatite ^29^.

Cention N contains organic monomer in the liquid consisting of a combination of UDMA, which is a hydrophobic agent of high viscosity, DCP (Tricyclodecane dimethanol dimethacrylate) and PEG-400 DMA (Polyethylene glycol dimethacrylate), which gets interconnected during the light cure reaction. UDMA is the main component of the monomeric matrix. Isofiller, which is a patented silane-functionalized filler material, is bonded to other material particles which improve the bond between the organic monomer matrix and the inorganic filler ^30^. Cention N in light cure mode showed higher bond strength than in non-light cured mode probably due to the dense polymer network formation. The same must not have occurred when it was not polymerized. It was not possible to measure the bond strength, which may also be related to the greater solubility, probably hindered polymerization at the interface.

In this study, the fact that the materials did not undergo the aging procedure can be considered as a limitation. This could lead to closer results between the groups when in oral function. However, it was possible to compare these new materials with the classical restorative materials.

In conclusion, the Equia Forte showed less solubility and presented higher microhardness which makes it a material a good alternative as a restorative material. Cention N showed a higher degree of conversion and high flexural strength making it an esthetic option to amalgam for posterior restoration because it has similar qualities to resin composite. However, the low bond strength values can limit the indication of these materials as restorative materials in the long term.

## References

[1] Tuzuner T, Dimkov A, Nicholson JW (2019). The effect of antimicrobial additives on the properties of dental glass-ionomer cements: a review. Acta Biomater Odontol Scand.

[2] Duarte ACA, Pereira R, Carvalho SM (2022). Enhancing glass ionomer cement features by using the calcium phosphate nanocomposite. Braz Dent J.

[3] Nogueira CHP, Gelio MB, Besegato JF (2023). Effect of aging and cementation systems on the bond strength to root dentin after fiber post cementation. Braz Dent J.

[4] Bhatia HP, Singh S, Sood S, Sharma N (2017). A Comparative Evaluation of Sorption, Solubility, and Compressive Strength of Three Different Glass Ionomer Cements in Artificial Saliva: An in vitro Study. Int J Clin Pediatr Dent.

[5] Poornima P, Koley P, Kenchappa M (2019). Comparative evaluation of compressive strength and surface microhardness of EQUIA Forte, resin-modified glass-ionomer cement with conventional glass-ionomer cement. J Indian Soc Pedod Prev Dent.

[6] Vallittu PK, Boccaccini AR, Hupa L, Watts DC (2018). Bioactive dental materials-Do they exist and what does bioactivity mean?. Dent Mater.

[7] Bramhill J, Ross S, Ross G (2017). Bioactive Nanocomposites for Tissue Repair and Regeneration: A Review. Int J Environ Res Public Health.

[8] Naoum S, Ellakwa A, Martin F, Swain M (2011). Fluoride release, recharge and mechanical property stability of various fluoride-containing resin composites. Oper Dent.

[9] Ong J, Yap AU, Abdul Aziz A, Yahya NA (2023). Flexural Properties of Contemporary Bioactive Restorative Materials: Effect of Environmental pH. Oper Dent.

[10] ISO 4049 (2019). Dentistry - Polymer-based restorative materials. International Standard Organization.

[11] da Silva RC, Zuanon AC, Spolidorio DM, Campos JA (2007). Antibacterial activity of four glass ionomer cements used in atraumatic restorative treatment. J Mater Sci Mater Med.

[12] Gavranović-Glamoč A, Ajanović M, Korać S (2017). Evaluation of the water sorption of luting cements in different solutions. Acta Med Acad.

[13] Tavangar MS, Jafarpur D, Bagheri R (2017). Evaluation of compressive strength and sorption/solubility of four luting cements. Journal of dental biomaterials.

[14] Matick ACC, Navarro CH, Higashi DT (2019). Evaluation of solubility and sorption in water of some direct restorative materials. Revista de Odontologia da UNESP.

[15] Todd J (2016). Scientific Documentation: Cention N. Ivoclar-Vivadent Press: Schaan.

[16] Farias JFGd, Andrade AKM, Duarte RM (2018). Water sorption and solubility of glass ionomer cements indicated for atraumatic restorative treatment considering the time and the pH of the storage solution. RGO-Revista Gaúcha de Odontologia.

[17] Mishra A, Singh G, Singh S (2018). Comparative evaluation of mechanical properties of Cention N with conventionally used restorative materials-an in vitro study. International Journal of Prosthodontics and Restorative Dentistry.

[18] Moshaverinia M, Navas A, Jahedmanesh N (2019). Comparative evaluation of the physical properties of a reinforced glass ionomer dental restorative material. J Prosthet Dent.

[19] Chole D, Shah HK, Kundoor S (2018). In vitro comparison of flexural strength of cention-n, bulkFill composites, light-cure nanocomposites and resin-modified glass ionomer cement. J Dent Med Sci.

[20] Balagopal S, Nekkanti S, Kaur K (2021). An In Vitro Evaluation of the Mechanical Properties and Fluoride-releasing Ability of a New Self-cure Filling Material. J Contemp Dent Pract.

[21] Fronza BM, Rueggeberg FA, Braga RR (2015). Monomer conversion, microhardness, internal marginal adaptation, and shrinkage stress of bulk-fill resin composites. Dental materials.

[22] Soh M, Yap AU (2004). Influence of curing modes on crosslink density in polymer structures. Journal of dentistry.

[23] Bolaños-Carmona V, Benavides-Reyes C, González-López S, González-Rodríguez P, Álvarez-Lloret P (2020). Influence of Spectroscopic Techniques on the Estimation of the Degree of Conversion of Bulk-fill Composites. Oper Dent.

[24] Chowdhury D, Guha C, Desai P (2018). Comparative evaluation of fracture resistance of dental amalgam, Z350 composite resin and cention-N restoration in class II cavity. IOSR J Dent Med Sci.

[25] Komandla DR, Acharya SR, Pentapati KC (2021). Comparative Evaluation of Surface Roughness of Resin-Modified Glass Ionomer and Glass Hybrid Restorative Materials Simulated by Tooth Brushing: An in-Vitro Study.

[26] Calvo AFB, Kicuti A, Tedesco TK, Braga MM, Raggio DPJBor (2015). Evaluation of the relationship between the cost and properties of glass ionomer cements indicated for atraumatic restorative treatment.

[27] Feiz A, Amrollahi N, Ziayi FJIJoP (2019). Comparative evaluation of microtensile bond strength of four glass-containing materials with primary teeth dentin.

[28] Manuja N, Pandit I, Srivastava N (2011). Comparative evaluation of shear bond strength of various esthetic restorative materials to dentin. An in vitro study.

[29] Manuja N, Pandit IK, Srivastava N, Gugnani N, Nagpal R (2011). Comparative evaluation of shear bond strength of various esthetic restorative materials to dentin: an in vitro study. J Indian Soc Pedod Prev Dent.

[30] Samanta S, Das UK, Mitra A (2017). Comparison of microleakage in class V cavity restored with flowable composite resin, glass ionomer cement and cention N. Imp J Interdiscip Res.

